# Pathogenesis and New Therapeutic Targets of Ovarian Cancer

**DOI:** 10.1155/2012/867512

**Published:** 2012-08-30

**Authors:** Ie-Ming Shih, Chih-Ming Ho, Kentaro Nakayama, Ritu Salani

**Affiliations:** ^1^Departments of Pathology, Oncology and Gynecology/Obstetrics, Johns Hopkins University School of Medicine, Baltimore, MD 21231, USA; ^2^Gynecologic Cancer Center, Department of Obstetrics and Gynecology, Cathay General Hospital, Jen-Ai Road, Taipei 10630, Taiwan; ^3^Department of Obstetrics and Gynecology, Shimane University School of Medicine, Enyacho 89-1, Izumo 693-8501, Japan; ^4^Division of Gynecologic Oncology, Department of Obstetrics and Gynecology, The Ohio State University, Comprehensive Cancer Center, Arthur G. James Cancer Hospital and Richard J. Solove Research Institute, Columbus, OH 43210, USA

Ovarian cancer remains the most lethal gynecologic malignancy, largely due to the lack of early detection tools and effective therapeutic interventions. Anti-tumor agents targeting critical molecular pathways hold promise for improving survival in these patients and understanding the critical molecular pathways involved in the pathogenesis of ovarian cancer development is central to the development of such agents. For example, using genome-wide DNA copy number analysis, investigators have identified amplification in a genomic locus (ch11q13.5) harboring a chromatin remodeling gene, RSF1, encoding Rsf-1 in high-grade ovarian serous carcinomas. Recent studies have shown that excessive Rsf-1 expression attributes to genomic instability and alters gene expression profiles to favor tumor growth and survival, especially in the presence of cytotoxic agents. The article entitled “*DNA damage response is prominent in ovarian high-grade serous carcinomas, especially those with Rsf-1 (HBXAP) overexpression*” reported by M. Kushirsagar et al. in this special issue provides new evidence that Rsf-1 overexpression was correlated with DNA damages which was observed more frequently in high-grade ovarian serous carcinoma. This finding should have several biological and clinical implications for the future studies of Rsf-1 in human cancer.

The article entitled “*Regulatory T cells in human ovarian cancer*” from D.-J. Peng et al. is a succinct review of the roles of the immune system in ovarian cancer development. In this article, they focus on summarizing the functions of regulatory T cells in the pathogenesis of ovarian cancer from a perspective of immune response and regulation. The knowledge of ovarian cancer immunity is fundamental to understand the molecular interplay between cancer cells and their tumor microenvironment and serves as an important road map for future development of immune-based therapy in ovarian cancer.

 Another interesting review article in this special issue is from C. Ohyagi-Hara et al. who summarized the potential to apply *integrin inhibitors as a therapeutic agent for ovarian cancer.* This is considered as a highly rational approach because the initial critical step of ovarian cancer metastasis is the attachment of cancer cells onto the peritoneum surface, and targeting integrins holds promise to inhibit ovarian cancer metastasis. Although no integrin inhibitors have shown favorable results so far, integrin-targeted therapies remain attractive for further clinical investigation.

The review article “*Optimizing molecular targeted therapies in ovarian cancer: the renewed surge of interest in ovarian cancer biomarkers and cell Signaling pathways*” from D. Hiss timely reviewed the biomarkers and signaling pathways of ovarian cancer. The author comprehensively summarized the recent advances in this highly competitive field with special emphasis on their translational implications. The number of literatures cited exceeds 400 which provide a compendium for ovarian cancer biomarker studies. The review article entitled “*Ovarian cancer: opportunity for targeted therapy*” by T. Tagawa et al. focuses on discussing exciting molecular targets including PARP, MEK, microRNAs, and molecules involved in angiogenesis. The authors keenly separate different types of ovarian cancer (type I and type II) in the discussion because it has become increasingly clear that different histological subtypes of ovarian cancer use distinct molecular alterations for their development. Thus, it is essential to consider this important factor in studying molecular targeting and developing new therapeutics in ovarian cancer. To this end, the article “*GRP78 expression in ovarian cancer patients and perspectives for a drug-targeting approach”* proposes to use GRP78 as a drug delivery system targeting ovarian cancer cells. This is of great interest given that GRP78 upregulation is considered as a cellular response to endoplasmic reticulum stress which is common in tumor cells. The finding of abundant GRP78 molecules in ovarian cancer cell surface invites the development of novel drug delivery to specifically bring the cytotoxic drug or other antitumor agents in the future clinical test. Another very interesting article “*Special agents hunting down women silent killer: the emerging role of the p38*α* kinase*” described the potential to target the pathways involved in cancer-specific metabolism and drug resistance. One of the pathways that the authors highlighted is the p38alpha which has been in the cancer research spotlight in recent years. Moreover, small compound inhibitors of p38alpha have been evaluated in clinical studies and the encouraging results should hold promise for the future applications of p38 inhibitors as an emerging treatment option for ovarian cancer treatment.

Finally, as tumor imaging techniques have advanced significantly in the past several years, the article entitled “*Early detection of ovarian cancer with conventional and contrast-enhanced transvaginal sonography: recent advances and potential improvements*” is a timely review that summarizes the potential to use imaging systems to early detect ovarian cancer and distinguish benign versus malignant ovarian neoplasms. For example, it has been shown that the 3D-transvaginal sonography combined with matrix array transducers/probes can enhance the visual inspection of cystic wall anomalies in adnexal masses, promote the comfort for patients and most importantly, improve reproducibility. In summary, we hope that these articles appearing in this special issue will provide useful research information to those investigators who are devoted to ovarian cancer research in the years to come.

*Ie-Ming Shih* *Ie-Ming Shih* *Chih-Ming Ho* *Chih-Ming Ho* *Kentaro Nakayama* *Kentaro Nakayama* *Ritu Salani* *Ritu Salani*

